# The Approved Live-Attenuated Chikungunya Virus Vaccine (IXCHIQ^®^) Elicits Cross-Neutralizing Antibody Breadth Extending to Multiple Arthritogenic Alphaviruses Similar to the Antibody Breadth Following Natural Infection

**DOI:** 10.3390/vaccines12080893

**Published:** 2024-08-07

**Authors:** Whitney C. Weber, Zachary J. Streblow, Craig N. Kreklywich, Michael Denton, Gauthami Sulgey, Magdalene M. Streblow, Dorca Marcano, Paola N. Flores, Rachel M. Rodriguez-Santiago, Luisa I. Alvarado, Vanessa Rivera-Amill, William B. Messer, Romana Hochreiter, Karin Kosulin, Katrin Dubischar, Vera Buerger, Daniel N. Streblow

**Affiliations:** 1 Vaccine and Gene Therapy Institute, Oregon Health and Science University, Beaverton, OR 97006, USA; weberw@ohsu.edu (W.C.W.); strebloz@ohsu.edu (Z.J.S.); kreklywi@ohsu.edu (C.N.K.); dentonm@ohsu.edu (M.D.); sulgey@ohsu.edu (G.S.);; 2 Department of Molecular Microbiology and Immunology, Oregon Health and Science University, Portland, OR 97239, USA; messer@ohsu.edu; 3 Ponce Research Institute, Ponce Health Sciences University, Ponce 00716, Puerto Rico; dmarcano@psm.edu (D.M.); pflores21@stu.psm.edu (P.N.F.); rmrodriguez@psm.edu (R.M.R.-S.); lalvarado@psm.edu (L.I.A.); vrivera@psm.edu (V.R.-A.); 4 Valneva Austria GmbH, 1030 Vienna, Austria; romana.hochreiter@valneva.com (R.H.); karin.kosulin@valneva.com (K.K.); katrin.dubischar@valneva.com (K.D.); vera.buerger@valneva.com (V.B.); 5 Division of Pathobiology and Immunology, Oregon National Primate Research Center, Beaverton, OR 97006, USA

**Keywords:** chikungunya virus (CHIKV), alphaviruses, IXCHIQ, vaccine, cross-neutralization, antibody, breadth, infection

## Abstract

The first vaccine against chikungunya virus (CHIKV) was recently licensed in the U.S., Europe, and Canada (brand IXCHIQ^®^, referred to as VLA1553). Other pathogenic alphaviruses co-circulate with CHIKV and major questions remain regarding the potential of IXCHIQ to confer cross-protection for populations that are exposed to them. Here, we characterized the cross-neutralizing antibody (nAb) responses against heterotypic CHIKV and additional arthritogenic alphaviruses in individuals at one month, six months, and one year post-IXCHIQ vaccination. We characterized nAbs against CHIKV strains LR2006, 181/25, and a 2021 isolate from Tocantins, Brazil, as well as O’nyong-nyong virus (ONNV), Mayaro virus (MAYV), and Ross River virus (RRV). IXCHIQ elicited 100% seroconversion to each virus, with the exception of RRV at 83.3% seroconversion of vaccinees, and cross-neutralizing antibody potency decreased with increasing genetic distance from CHIKV. We compared vaccinee responses to cross-nAbs elicited by natural CHIKV infection in individuals living in the endemic setting of Puerto Rico at 8–9 years post-infection. These data suggest that IXCHIQ efficiently and potently elicits cross-nAb breadth that extends to related alphaviruses in a manner similar to natural CHIKV infection, which may have important implications for individuals that are susceptible to alphavirus co-circulation in regions of potential vaccine rollout.

## 1. Introduction

Chikungunya virus (CHIKV) is a human pathogenic alphavirus responsible for sporadic epidemics that have burdened 100+ countries over 50 years. From 2013 to 2023, there were over 3.68 million suspected and confirmed cases in 50 countries in the Americas [1]. Alphaviruses are part of the *Togaviridae* family composing a number of additional emerging human pathogenic viruses that are predominantly mosquito-transmitted. CHIKV belongs to the Semliki Forest antigenic complex, which includes seven additional viruses with varying degrees of cross-reactivity due to shared antigenicity. While there are three distinct lineages of CHIKV, Asian lineage, East Central South African (ECSA) lineage, and West African lineage, a fourth Indian Ocean lineage (IOL) has been proposed to exist. Immunologically, these lineages conform to a single serotype [2,3]. It has been observed that even decades after large CHIKV outbreaks, herd immunity limits additional outbreaks or the emergence of new CHIKV strains in that region, further supporting a single serotype [3]. Predominantly circulating CHIKV strains have yet to accumulate mutations to confer host antibody-neutralization escape, but the viral evolution of CHIKV continues to be cause for concern after single mutations have conferred transmissibility in new mosquito vectors [4]. This warrants investigation to identify differences in CHIKV antibody potency against diverse strains. Notable emerging viruses that have contributed to sporadic outbreaks within the Semliki Forest antigenic complex include O’nyong-nyong virus (ONNV), Mayaro virus (MAYV), and Ross River virus (RRV). Each of these viruses has been shown to co-circulate with CHIKV [5,6,7], and they are transmitted by similar vectors as several flaviviruses such as dengue and Zika viruses [8]. This leads to co-circulation of diverse human pathogenic arbovirus infections, presenting urgent public health concern.

Vaccines to counter CHIKV have been in development for decades based on virus-like particle, viral vector, live-attenuated virus, nucleic acid, subunit, and inactivated viral vaccine platforms. The primary goal of these vaccine platforms has been to elicit high levels of neutralizing antibodies, which are generally accepted as the main correlate of protection, although protective roles of T cells have also been established [9,10]. There are several CHIKV vaccines currently in clinical trials, with two vaccines in Phase I, two vaccines in Phase II, and two vaccines in Phase III trials [11]. In November of 2023, the U.S. Food and Drug Administration (FDA) approved the CHIKV vaccine IXCHIQ (VLA1553), which was a huge step for the alphavirus field, especially amidst a year with over 500,000 CHIKV cases, with the epicenter in Brazil, where many additional arboviruses burden the community. The European Medicines Agency has also now officially approved marketing authorization of IXCHIQ in the European Union [12]. Under the OPEN regulatory procedure, this review was joined by other regulators including Brazilian ANVISA, marking the first endemic country reviews [13]. The IXCHIQ vaccine is a single-dose, live-attenuated vaccine (LAV) platform based upon the CHIKV_LR2006-OPY1_ backbone containing a large genetic deletion in nsP3 [14]. IXCHIQ was tested in mice and cynomolgus macaques to establish a protective antibody threshold due to the challenges of conducting an efficacy trial given the sporadic nature of CHIKV outbreaks [14,15,16,17]. IXCHIQ has now been tested in over 4000 individuals in non-endemic settings and is generally immunogenic and well tolerated, although viremia and some side effects including headache, fever, arthralgia, and myalgia have been noted [18,19,20]. Additional trials are ongoing to evaluate IXCHIQ in endemic settings, in adolescents, and to examine long-term antibody persistence. A study is planned in moderately immunocompromised individuals living with HIV.

IXCHIQ approval brings forward many scientific questions regarding the breadth of cross-reactive immunity and the implications this may have in communities with the potential of alphavirus co-circulation. It is well established that primary CHIKV infection in humans has the ability to elicit cross-neutralizing immune responses that extend to related alphaviruses within the Semliki Forest virus (SFV) complex, which is driven, in large part, by antibodies recognizing the envelope protein E2 domain B region [21,22,23]. Although live-attenuated vaccines are capable of causing symptoms similar to infection, there is a clear benefit to eliciting robust immune responses with potency similar to a natural infection. For the purposes of this study, we sought to quantify neutralizing antibody responses in IXCHIQ vaccinees and participants with CHIKV infection across CHIKV genotypes and to other members of the SFV complex including ONNV, MAYV, and RRV. We anticipated that the neutralizing antibody potency and breadth would be similar to the immunity elicited by natural CHIKV infection due to the live-attenuated nature of the vaccine. Our data demonstrate a comparable antigenic profile induced by IXCHIQ immunization and natural CHIKV infection, offering promising implications for alphavirus cross-protection.

## 2. Materials and Methods

### 2.1. Ethics Statement

All participants gave their informed consent for inclusion before they participated in this study. This study was conducted in accordance with the Declaration of Helsinki and approved by the central Institutional Review Board (Advarra IRB #Pro00045587, approved 6 August 2020, and #Pro00050546, approved 24 March 2021) for the human IXCHIQ vaccinees in the NCT04546724 and NCT04838444 clinical trials. The study has been reviewed and approved by the Oregon Health and Science University (OHSU) IRB (IRB #10212, 6 November 2015) for the CHIKV endemic cohort.

### 2.2. Study Participants

IXCHIQ recipient vaccinee cohort

A total of 120 samples from 30 adult participants that were part of VLA1553 clinical trials in the continental U.S., NCT04546724 and NCT04838444, were included in this exploratory analysis. Samples were selected based on availability and based on neutralization titers measured in the clinical trial to represent an average neutralization capacity. Samples were also selected with intent to represent heterogeneity in a population, with specific attention to age and sex of the participants. All participants were considered generally healthy and had no documented history of CHIKV infection or arthralgia.

Endemic cohort participant samples collected after CHIKV infection in Puerto Rico

Individuals with CHIKV infection histories in this study were enrolled in a larger study of long-term immunity following infection with arthropod-borne viruses. Samples were provided by Dr. Vanessa Rivera-Amill (Ponce Health Sciences University, Ponce, Puerto Rico). Samples from participants in this study with either PCR-confirmed CHIKV infections or that had positive CHIKV IgG/IgM ELISA results were screened for CHIKV neutralizing antibodies. Nine samples had detectable neutralizing activity, and these were included in this study. Following informed consent, participants provided additional history including other known and suspected arboviral infections, lifetime travel histories, and vaccination histories. Samples were collected, processed, and shipped to Oregon Health & Science University for analysis.

### 2.3. Cells and Viruses

Vero cells (ATCC CCL-81) were cultured in Dulbecco’s Modified Eagle Medium (DMEM; Corning, Manassas, VA, USA) containing 5% fetal calf serum (FCS; Thermo Scientific, Waltham, MA, USA) supplemented with 1× penicillin–streptomycin–glutamine (PSG; Life Technologies, Carlsbad, CA, USA) (DMEM-5). *Aedes albopictus* C6/36 cells (ATCC CRL1660) were also cultured in DMEM-5. Vero cells were propagated at 37 °C and 5% CO_2_ and C6/36 cells were propagated at 28 °C with 5% CO_2_. Alphaviruses were sourced through the Biodefense and Emerging Infections Research Resources Repository (BEI Resources, Manassas, VA, USA): MAYV_BeAr505411_ (BEI NR-49910), ONNV_UgMP30_ (BEI NR-51661), and RRV_T-48_ (BEI NR-51457). The infectious clone of CHIKV_LR2006-OPY1_ was provided by Steven Higgs (Kansas State University, Manhattan, KS, USA). The infectious clone of CHIKV_181/25_ was provided by Terence Dermody (University of Pittsburgh, Pittsburgh, PA, USA). The CHIKV_Brazil-7124_ infectious clone was engineered as described below. Viral stocks were generated from the two infectious CHIKV clones as previously described [9]. Alphaviruses were propagated in *Aedes albopictus* C6/36 cells. Cell culture supernatants were harvested and clarified 72 h post-infection (hpi) then pelleted through a 10% sorbitol cushion by ultracentrifugation at 82,755× *g* for 70 min. The viral pellets were resuspended in PBS, frozen at −80 °C, and titered by limiting dilution plaque assays using confluent monolayers of Vero cells. Infected cells were rocked continuously for 2 h at 37 °C with 5% CO_2_ and overlaid with a 2:1 mixture of DMEM-5 to 0.3% high-/0.3% low-viscosity carboxymethyl cellulose (Sigma-Aldrich, St. Louis, MO, USA). Plaque assays were fixed with 3.7% formaldehyde and stained with 0.2% methylene blue at 48 hpi for CHIKV_LR2006_, CHIKV_Brazil-7124_, MAYV_BeAr505411_ and RRV_T-48_ or 72 hpi for CHIKV_181/25_ and ONNV_UgMP30_. Plaques were visualized and enumerated using a dissecting microscope and the counts were used to calculate viral titers in plaque-forming units per mL. Virus stocks used for all described experiments were either passage 1 or 2 and were sequence-validated as described below.

### 2.4. Construction of the CHIKV Brazil Infectious Clone

To assemble the infectious clone of chikungunya virus strain TO-UFT-7124 (CHIKV_Brazil-7124_), seven genome fragments of approximately 1700 bp, each with 20 bp of overlapping sequence, were synthesized by Twist Bioscience using the sequence originally reported [24]. The approximately 2200 bp vector was amplified by PCR with 20 bp of overlapping sequence with fragment 1 and fragment 7 under standard PCR conditions. Each fragment (200 fmol) was combined with an equal volume of NEBuilder HiFi master mix (NEB, Ipswich, MA, USA) according to the manufacturer’s instructions. Assembly was performed at 50 °C for 60 min. TOP10 competent cells (Invitrogen, Waltham, MA, USA) were transformed with 5 µL of the assembled PCR product. After DNA purification, the CHIKV_Brazil-7124_ infectious clone was verified by whole plasmid sequencing (Eurofins, Louisville, KN, USA). The CHIKV_Brazil-7124_ plasmid was linearized with *Not* I digestion and transcribed in vitro with the SP6 mMessage mMachine kit (Invitrogen). cDNA clone-derived RNA was purified using the RNeasy Mini Kit (Qiagen, Hilden, Germany). Vero cells were transfected with 10 µg of RNA and 6 µL of Lipofectamine 2000 per well of a 6-well plate, according to the Invitrogen protocol. At 72 h post-infection, supernatant was harvested and stored at −80 °C. Virus stocks were propagated with 100 µL of resulting p0 per T-175 flask of C6/36 cells and purified as described above.

### 2.5. Next-Generation Sequencing of Viral Stocks

Next-generation sequencing (NGS) was used to sequence-verify the viruses used in this study. Vero cells cultured in DMEM-5 were added to 6-well plates and incubated overnight at 37 °C, 5% CO_2_. For each viral infection, 100 µL of virus stock for CHIKV_LR2006-OPY1_ (accession DQ443544.2), CHIKV_181/25_ (accession MW473668.1), CHIKV_Brazil-7124_ (accession ON586955.1), MAYV_BeAr505411_ (accession KP842818.1), ONNV_UgMP30_ (accession M20303.1), and RRV_T-48_ (accession GQ433359.1) was added to 6 mL of DMEM-5, mixed, and distributed between 3 wells of a 6-well plate. Cells were incubated at 37 °C with 5% CO_2_ and were allowed to infect for 24 h. Cell supernatants were removed; cells were resuspended in 1 mL of Trizol reagent per well, incubated at room temperature for 5 min, and collected with a cell scraper and transferred into new tubes for processing. RNA was extracted from Trizol-suspended samples following the manufacturer’s protocol and eluted in 20 µL of dH_2_O. The concentration and quality of RNA were determined by OD using a Thermo Scientific Nanodrop instrument. NGS libraries were produced with Illumina’s Stranded mRNA Prep kit and qualified with Agilent’s Bioanalyzer 2000. Viral reads from RNA sequencing data were compared to the corresponding GenBank reference viral sequences listed above using Geneious Prime version 2024.0.3 software. Results listing nucleotide and amino acid changes and positions are shown in Supplemental Table S1. To construct the maximum likelihood phylogenetic tree in MEGA version 11.0.13 software, we first trimmed the sequences to focus on the structural proteins E1/6K/E2/E3, wherein the majority of the neutralizing antibody epitopes reside.

### 2.6. Neutralization Assays (50% Plaque Reduction Neutralization Test, PRNT_50_)

Neutralization assays were performed as previously described [21]. Briefly, serum samples from 30 human vaccinee participants at four timepoints (baseline and 1 month, 6 months, and 1 year post-vaccination with IXCHIQ) and from 9 CHIKV-immune individuals at ~8 and ~9 years post-infection were tested for neutralization activity against six viruses: CHIKV_181/25_, CHIKV_LR2006-OPY1_, CHIKV_Brazil-7124_, MAYV_BeAr505411_, ONNV_UgMP30_, and RRV_T-48_. Serum was heat-inactivated for 30 min at 56 °C. Vero cells were cultured in 12-well plates in DMEM-5 plated one day prior to infection to reach confluency. The following day, serum was serially diluted in DMEM-5 starting at 1:10 in 96-well U-bottom plates, and further diluted for a total of 11 2-fold dilutions. Each virus was diluted to a concentration that generated 50–200 plaques per well in DMEM-5 and added to 96-well round-bottom plates. Equivalent volumes of each diluted serum series were transferred and mixed in the plates containing the diluted virus then rocked continuously and incubated at 37 °C with 5% CO_2_ for 2 h. After 2 h, 200 µL of each serum/virus mixture was used to infect each well of a 12-well plate. Plates were rocked continuously and incubated at 37 °C with 5% CO_2_ for 2 h. Cells were overlaid with a 2:1 mixture of DMEM-5 to 0.3% high-/0.3% low-viscosity carboxymethyl cellulose and incubated at 37 °C for 48 h (72 h for CHIKV_181/25_ ONNV_UgMP30_). After incubation, cells were fixed with 3.7% formaldehyde and stained with 0.2% methyl blue. Plaques were manually counted under a microscope or by eye to determine the percentage of plaques at each dilution relative to the number of plaques in virus-only (no serum) control wells. Finally, 50% plaque reduction neutralization titers (PRNT_50_) were calculated by variable slope non-linear regression in GraphPad Prism version 10 software. PRNT_50_ values are reported as the inverse serum dilution. Baseline negative control human serum samples (day 1) were run in every experiment.

### 2.7. Antigenic Cartography

Antigenic cartography was used to visualize the serologic relationships between vaccinee and naturally infected patient samples and the profile of neutralized viruses based on 50% plaque reduction neutralization titers (PRNT_50_) as previously described [25,26]. Maps were constructed using the Racmacs package (https://acorg.github.io/Racmacs/, version 1.1.35., accessed on 10 April 2024) in R (version 2023.12.1+402). For serum with neutralization under the limit of detection, the PRNT_50_ was recorded as 10 for all calculations. The map was computed for 500 optimizations with two dimensions, and the minimum column basis was set to none. The Bayesian method was used to perform 1000 bootstrap repeats with 100 optimizations per repeat of the PRNT_50_ data with the standard deviation of measurement noise set at 0.7 for both antigen and titer. Finally, blobs were added to the map using the “ks” algorithm method to represent the confidence levels of the neutralized viruses with the confidence level set at 0.68 and grid spacing set at 0.1. Each box of the grid is representative of a 2-fold difference in serum dilution.

### 2.8. Statistical Analysis

All statistical analysis was completed using GraphPad Prism version 10 software. Normalized variable slope non-linear regressions with upper and lower limits of 100 and 0, respectively, were used to calculate 50% plaque reduction neutralization titers (PRNT_50_). For age and PRNT_50_ correlations, both age and PRNT_50_ were first log-transformed before applying Pearson correlation and simple linear regressions. For the correlation of PRNT_50_ and Dayhoff distance, Spearman correlation was used.

## 3. Results

### 3.1. IXCHIQ Elicits Broad Alphavirus Immunity against CHIKV Strains as Well as Related ONNV, MAYV, and RRV

We sought to assess the presence of alphavirus cross-neutralizing antibodies at day 1 (baseline), day 29 (expected peak of response), day 180 (expected setpoint level), and day 365 (durability assessment) post-vaccination in human sera from 30 adult vaccinees aged 19 to 71 who were immunized with IXCHIQ (VLA1553 prior to approval) in clinical trial NCT04546724 and included in the follow-up trial NCT04838444 (Table 1). Samples from 17 females and 13 males with an age range of 19 to 76 with a median of 42.5 were tested (Table 1). We propagated a panel of six viruses within the Semliki Forest antigenic complex including three CHIKV strains (LR2006, 181/25, Brazil-7124), ONNV_UgMP30_, MAYV_BeAr505411_, and RRV_T-48_. The panel of alphaviruses were propagated from viral stocks provided by BEI or infectious clones provided by generous collaborators. CHIKV Brazil strain 7124 of the ESCA genotype, a contemporary 2021 Brazilian isolate, was generated using Gibson assembly methods based on the sequence of sample TO-UFT-7124 collected in 2021 in Tocantins, Brazil [24]. We included CHIKV_LR2006_ because the IXCHIQ live-attenuated vaccine was derived from the CHIKV LR2006-OPY1 strain from the ECSA genotype. We also included the attenuated CHIKV_181/25_ strain of the Asian genotype derived from the strain AF15561 as 181/25 is a previously characterized vaccine virus that was discontinued after Phase II clinical trials [27]. Additionally, the 181/25 strain was used in neutralization assays performed during Phase III clinical trials of VLA1553. ONNV_UgMP30_ is both phylogenetically and antigenically highly similar to CHIKV and has caused sporadic human outbreaks in sub-Saharan Africa. MAYV_BeAr505411_ and RRV_T-48_ are pathogenic, clinically relevant alphaviruses with the ability to cause outbreaks and human disease and circulate in South America and Australia, respectively. The alphavirus stocks were sequence verified using next-generation sequencing of the viral genomes and the mutations are listed in Supplemental Table S1. The detected mutation levels are considered low, and because most of the mutations were in the non-structural proteins, they are not predicted to impact antibody neutralization epitopes that are dominantly located in the structural proteins.

**Figure 1:**
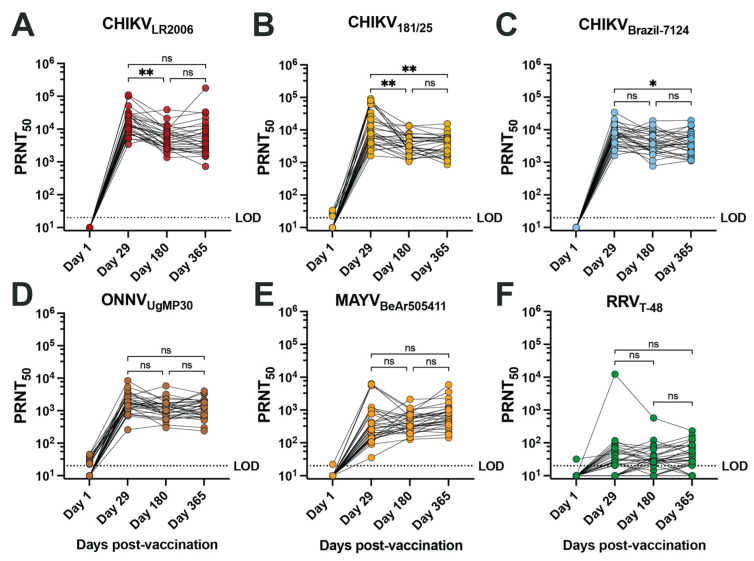
IXCHIQ immunization of human participants elicits antibodies that neutralize multiple CHIKV strains and cross-neutralize related arthritogenic alphaviruses. Serum from 30 human vaccinees was collected at days 1, 29, 180, and 365 after administration of the IXCHIQ vaccine and used in 50% plaque reduction neutralization titer assays (PRNT_50_) performed on Vero cells. Neutralizing antibody titers calculated using variable slope non-linear regressions in Prism software are shown in logarithmic scale for neutralization against (**A**) CHIKV_LR2006_, (**B**) CHIKV_181/25_, (**C**) CHIKV_Brazil-7124_, (**D**) ONNV_UgMP30_, (**E**) MAYV_BeAr505411_, and (**F**) RRV_T-48_. Repeated-measure one-way ANOVA with Geisser–Greenhouse correction was used to compare PRNT_50_ values among day 29, 180, and 365 timepoints. A Bonferroni test to correct for multiple comparisons was performed where ns *p* > 0.05, * *p* = 0.0177, and ** *p* < 0.001. The limit of detection for the assay was 20, and sera falling below the limit of detection were assigned a titer of 10.

To assess the neutralizing antibody potency and breadth of the vaccinee sera, we performed 50% plaque reduction neutralization tests (PRNT_50_) on confluent monolayers of Vero cells for serum samples collected at days 1, 29, 180, and 365 post-vaccination from 30 vaccinees (Figure 1, Table 2 and Supplemental Table S2). Overall, seroconversion status (PRNT_50_ ≥ 20) was reached for 100% of vaccinees for each of the three CHIKV strains, ONNV_UgMP30_, and MAYV_BeAr505411_ as early as day 29 post-vaccination and was maintained at day 180 and day 365 post-vaccination (Table 2). RRV_T-48_ was the only virus where seroconversion was not reached in all vaccinees, with seroconversion rates reduced to 66.6% at day 29, 83.3% at day 180, and 83.3% at day 365 post-vaccination (Table 2). The neutralization titers against RRV are considered very low. Consistent with IXCHIQ (VLA1553) clinical trial immunogenicity findings where µPRNT testing was performed using attenuated heterologous strain CHIKV_181/25_ [18], we found that the homotypic PRNT_50_ geometric mean titer (GMT) against CHIKV_LR2006_ peaked at day 29 post-vaccination at 16,022 and gradually leveled off at a titer of 5147 at day 365 post-vaccination (Figure 1A, Table 2) [18,20]. Vaccinee GMT declined significantly between days 29 and 180 post-vaccination (** *p* = 0.0018) (Figure 1A). The kinetics of the antibody potency against CHIKV_181/25_ were similar with peak GMT at day 29 post-vaccination of 10,879 and a significant reduction to 3714 at days 180 (** *p* = 0.0054) and 3440 by day 365 post-vaccination (** *p* = 0.0048 compared to d29) (Figure 1B and Table 2). For the contemporary CHIKV_Brazil-7124_ strain, GMTs peaked at day 29 post-vaccination at 6491 and declined to 3881 by day 180 and 3792 by day 365; only the comparison between day 29 and day 365 reached statistical significance (* *p* = 0.0177) (Figure 1C and Table 2). This finding indicated that IXCHIQ immunization elicits neutralizing responses against a recently circulating CHIKV isolate. The IXCHIQ vaccine also elicited heterotypic neutralizing antibodies against ONNV_UgMP30_ that did not change significantly over time, with a peak GMT at day 29 of 1.676, 1102 at day 180, and 1156 at day 365 post-vaccination (Figure 1D and Table 2). In contrast to the GMTs against CHIKV strains and ONNV_UgMP30_ that peaked at day 29 post-vaccination, the MAYV_BeAr505411_ GMT gradually increased over time from 374 at day 29 to 417 at day 180 and 652 at day 365; this gradual increase was not statistically significant (Figure 1E and Table 2). Finally, for RRV_T-48_, which is the most distantly related Semliki Forest antigenic complex member included in our investigation, the GMT was 32 at day 29, 33 at day 180, and 39 at day 365 post-vaccination; there was consistency of these low-level responses over time and these changes did not reach statistical significance (Figure 1F and Table 2). Altogether, these data demonstrate that IXCHIQ immunization elicits both homotypic and heterotypic cross-neutralizing immunity extending to related arthritogenic alphaviruses in vaccine recipients. Importantly, both the homotypic and heterotypic neutralizing responses retained durability up to one year post-vaccination.

### 3.2. The Potency of Alphavirus Neutralizing Antibodies for IXCHIQ Vaccinees Decreases with Decreasing Genetic Similarity of Viral Antigens

**Figure 2:**
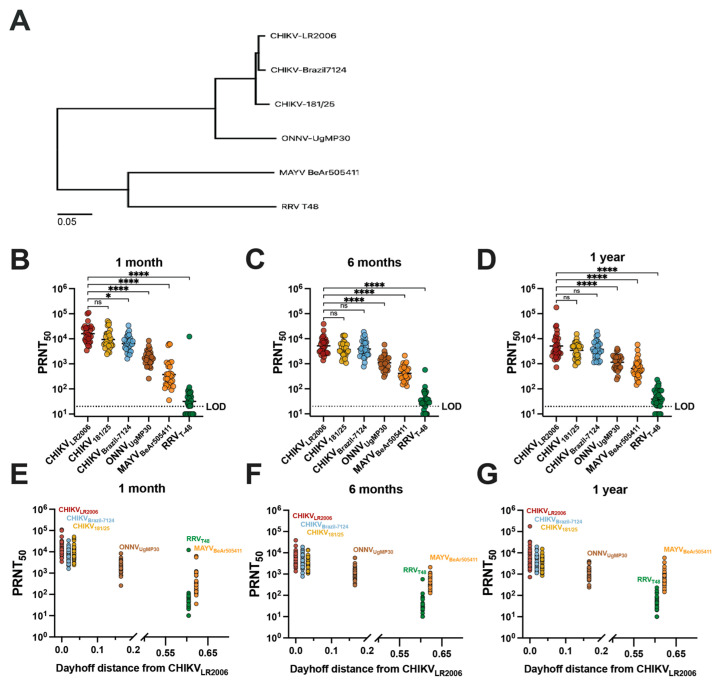
Cross-neutralizing antibodies decrease in potency with increasing phylogenetic and Dayhoff distance from the CHIKV_LR2006_ parental vaccine strain. The maximum likelihood phylogenetic tree shown in (**A**) for the viruses used in this study represents viral stock sequencing consensus data aligned and trimmed to E1/6K/E2/E3 in Geneious Prime version 2024.0.3 software with phylogeny constructed in MEGA version 11.0.13 software using the Dayhoff model with uniform rates and nearest-neighbor interchange. Neutralizing antibody titers for each virus by 50% plaque reduction neutralization test (PRNT_50_) are grouped on logarithmic scale by (**B**) 1-month, (**C**) 6-month, and (**D**) 1-year post-vaccination timepoints. Variable slope, non-linear regressions in Prism software were used to calculate PRNT_50_. Neutralizing activity is compared to the parental vaccine strain CHIKV_LR2006_ and data are analyzed by one-way ANOVA (Freidman’s test) with Dunn’s multiple comparisons where ns *p* > 0.05, * *p* = 0.0289, and **** *p* < 0.0001. Panels (**E**–**G**) represent analysis of serology data with Dayhoff distance of amino acid relatedness between each virus and the parental CHIKV_LR2006_ strain. Dayhoff distances were calculated using viral stock sequences trimmed to E1/6K/E2/E3 in MEGA version 11.0.13 software. The limit of detection for the neutralization assays was 20, and sera falling below the limit of detection were assigned a value of 10 for graphing and calculations of antigenic distances.

Next, we compared vaccinee serum homotypic and heterotypic neutralization titer results with the genetic relatedness of the neutralized viruses (Figure 2). To compare phylogenetic distances, we constructed a maximum likelihood phylogenetic tree based on the sequences of the structural proteins E1/6K/E2/E3 of the viruses included in our study (Figure 2A). We next grouped the neutralization data for IXCHIQ vaccinees by virus strain at 1 month (Figure 2B), 6 months (Figure 2C), and 1 year post-vaccination (Figure 2D) and found that the potency of neutralizing antibodies gradually decreased with increasing phylogenetic distance from the parental vaccine strain virus, CHIKV_LR2006_ (Figure 2A). At 1 month post-vaccination, compared to CHIKV_LR2006_, the GMT for CHIKV_181/25_ was ~1.4-fold lower (ns), ~2.4-fold lower for CHIKV_Brazil-7124_ (* *p* = 0.0289), ~9.6-fold lower for ONNV_UgMP30_ (**** *p* < 0.0001), ~43-fold lower for MAYV_BeAr505411_ (**** *p* < 0.0001), and ~505-fold lower for RRV_T-48_ (**** *p* < 0.0001) (Figure 2B). At 6 months post-vaccination, compared to CHIKV_LR2006_, the GMT for CHIKV_181/25_ was ~1.4-fold lower (ns), ~1.3-fold lower for CHIKV_Brazil-7124_ (ns), ~4.7-fold lower for ONNV_UgMP30_ (**** *p* < 0.0001), ~12.5-fold lower for MAYV_BeAr505411_ (**** *p* < 0.0001), and ~160-fold lower for RRV_T-48_ (**** *p* < 0.0001) (Figure 2C). At 1 year post-vaccination in comparison to CHIKV_LR2006_, the GMT against CHIKV strains and ONNV were nearly identical: CHIKV_181/25_ was ~1.2-fold lower (ns), CHIKV_Brazil-7124_ ~1.2-fold higher (ns), ONNV_UgMP30_ ~1.2-fold lower (**** *p* < 0.0001); greater differences were observed for MAYV_BeAr505411_ at ~5.1-fold-lower GMT (**** *p* < 0.0001) and ~112-fold lower for RRV_T-48_ (**** *p* < 0.0001) (Figure 2D). These data affirm the durability of cross-neutralizing antibodies up to one year post-vaccination (Figure 2B–D). The E1/6K/E2/E3 amino acid sequences were used to calculate Dayhoff distances (MEGA version 11.0.13 software) to compare the amino acid relatedness of our panel of viruses with respect to the most antigenic viral proteins in terms of antibody responses (Supplemental Table S3). The vaccinee neutralization data were grouped in the same manner by time post-vaccination but compared with the Dayhoff distance, revealing that decreasing cross-neutralizing antibody potency was related to increasing Dayhoff distance of the neutralized viruses (Figure 2E–G). Neutralizing antibody titer and Dayhoff distance were significantly negatively correlated by Spearman correlation (*p* < 0.0001, r = −0.7165) (Figure 2G). For example, RRV_T-48_ is the most genetically and antigenically divergent virus from CHIKV_LR2006_, resulting in the lowest serum neutralization capability. Indeed, this highlights that antibody epitope profiles induced by vaccination are dominantly located in the amino acids of these viral structural proteins and that small changes in Dayhoff distance have a significant impact on the neutralization titer (Figure 2E–G). Overall, by analyzing the vaccinee neutralization titers with respect to genetic relatedness, we were able to discern that the neutralizing antibody potency and breadth were related to the genetic similarity of the Semliki Forest antigenic complex viruses included in our investigation (Figure 2).

### 3.3. IXCHIQ Vaccinees Develop Alphavirus Cross-Neutralizing Antibody Potency and Breadth Similar to Individuals Who Were Naturally Infected with CHIKV

**Figure 3:**
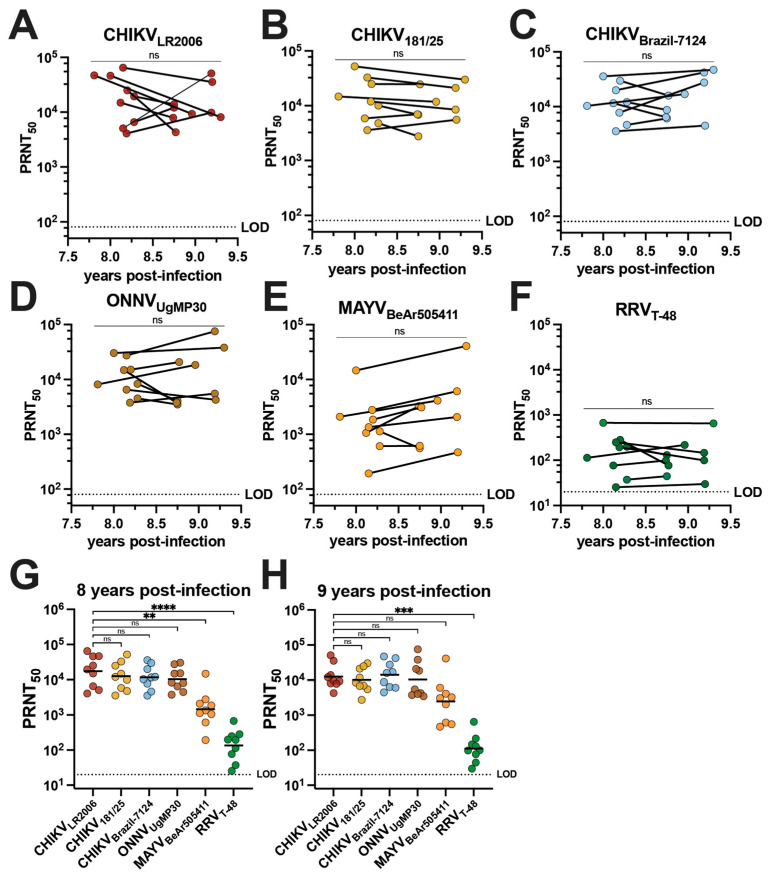
Neutralizing antibody breadth of human serum collected in Puerto Rico 8–9 years following 2014 CHIKV infections. Sera collected from nine human participants in Puerto Rico between June 2022 and November 2023 following diagnosed CHIKV infections in 2014 were used in virus neutralization assays against the six alphaviruses included in this study: (**A**) CHIKV_LR2006_, (**B**) CHIKV_181/25_, (**C**) CHIKV_Brazil-7124_, (**D**) ONNV_UgMP30_, (**E**) MAYV_BeAr505411_, and (**F**) RRV_T-48_. The PRNT_50_ data are plotted against year post-infection for each participant and the two timepoints (~8 and ~9 years post-infection) were compared by paired *t*-tests in (**A**–**F**). Neutralizing activity is also grouped by virus and strain at (**G**) ~8 years post-infection and (**H**) ~9 years post-infection. GMTs are shown in (**G**,**H**) where PRNT_50_ for CHIKV_LR2006_ is compared to PRNT_50_ for each other virus through analysis by one-way ANOVA (Freidman’s test) with Dunn’s multiple comparisons where ns *p* > 0.05, ** *p* = 0.0053, *** *p* = 0.002, and **** *p* < 0.0001. Variable slope, non-linear regressions in Prism software were used to calculate PRNT_50_. The limit of detection for neutralization assays was 20.

The cross-neutralizing antibody potency and breadth of vaccinee sera were compared to the sera of individuals who had experienced natural CHIKV infection during the 2014–2015 outbreak in Puerto Rico [28]. Serum samples were collected from nine adults aged 19–81 who had documented CHIKV infections in 2014 (Table 1). This group is composed of five female and four male participants who identify as Hispanic and were all born in Puerto Rico; the ethnicities of this group were Caucasian/white (*n* = 2), multiracial (*n* = 2), and other (*n* = 5) (Table 1). Although one individual was asymptomatic, the most common symptoms during infection were rash (77.7%), severe muscle/joint pain (66.6%), sensitivity to light (55.5%), and fever (55.5%) (Table 1). We determined the alphavirus cross-neutralizing antibody profiles for human sera from this cohort collected between June 2022 and November 2023 (Figure 3, Table 2 and Supplemental Table S4). There is no confirmation of which CHIKV strain was responsible for infection in these individuals, but the Asian genotype is known to have circulated in Puerto Rico at the time the patients were reportedly infected [29,30]. We detected potent neutralizing activity for all three CHIKV strains with no significant change in GMTs between the two timepoints (Figure 3A–C). GMTs of the cohort were observed for CHIKV_LR2006_ at 17,317 and 12,530 at ~8 and ~9 years post-infection, respectively (Figure 3A and Table 2). The GMTs for CHIKV_181/25_ were 12,522 and 10,146 at ~8 and ~9 years post-infection (Figure 3B and Table 2), and for CHIKV_Brazil-7124_, they were 11,639 and 14,138 at ~8 and ~9 years post-infection, respectively (Figure 3C and Table 2). Robust cross-neutralization was also observed for ONNV_UgMP30_ with GMTs of 10,370 at ~8 years post-infection and 10,360 at ~9 years post-infection with no significant change between the two timepoints (Figure 3D and Table 2). The cross-neutralizing antibody titers against MAYV_BeAr505411_ were 1462 at ~8 years post-infection and 2471 at ~9 years post-infection (Figure 3E and Table 2). The GMT for RRV_T-48_ was 135 at ~8 years post-infection and 112 at ~9 years post-infection (Figure 3F and Table 2). Importantly, 100% seroconversion against each alphavirus in this investigation was observed for these CHIKV-immune sera (Figure 3A–F and Table 2). At ~8 years post-infection, there were no significant differences when comparing the CHIKV_LR2006_ PRNT_50_ to CHIKV_181/25_, CHIKV_Brazil-7124_, and ONNV_UgMP30_ (Figure 3G). For the cross-neutralizing responses at ~8 years post-infection compared to CHIKV_LR2006_, the GMTs were ~11.8-fold lower against MAYV_BeAr505411_ (** *p* = 0.0053) and ~128-fold lower against RRV_T-48_ (**** *p* < 0.0001) (Figure 3G). At ~9 years post-infection, there were no significant changes when comparing the CHIKV_LR2006_ PRNT_50_ to CHIKV_181/25_, CHIKV_Brazil-7124_, ONNV_UgMP30_, and MAYV_BeAr505411_ (Figure 3H). The GMT was a significant ~112-fold lower for RRV_T-48_ at ~9 years post-infection (*** *p* = 0.002) (Figure 3H). Together, these data highlight that CHIKV infection induces cross-neutralizing breadth with no significant reduction in potency against the recently and potentially actively circulating contemporary CHIKV_Brazil-7124_ strain (Figure 3C). These findings also indicate the extension of neutralizing responses against the Semliki Forest antigenic complex, which remain stable for nearly a decade after infection (Figure 3).

**Figure 4:**
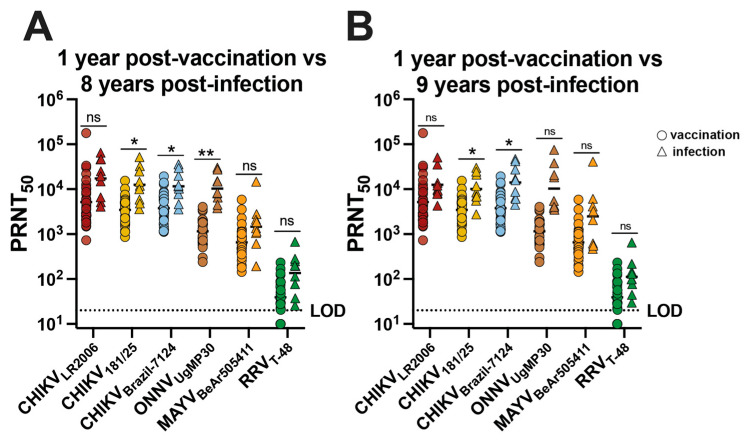
The neutralizing antibody breadth elicited by vaccination is comparable to CHIKV infection-induced cross-reactivity. Sera from vaccinees and from patients following CHIKV infection in Puerto Rico were used in virus plaque reduction neutralization assays against CHIKV_LR2006_, CHIKV_181/25_, CHIKV_Brazil-7124_, ONNV_UgMP30_, MAYV_BeAr505411_, and RRV_T-48_. The 50% plaque reduction neutralization titers (PRNT_50)_ were compared at 1 year post-vaccination with (**A**) ~8 years post-infection and (**B**) ~9 years post-infection. Variable slope, non-linear regressions in Prism software were used to calculate PRNT_50_. GMT in logarithmic scale is shown for each group. Data are analyzed by multiple unpaired t tests with Welch correction on each row where ns *p* > 0.05, * *p* < 0.05 and ** *p* < 0.001. The limit of detection for neutralization assays was 20, and sera falling below the limit of detection were assigned a value of 10.

The neutralizing antibody potency and breadth profiles were analyzed for CHIKV vaccinees and CHIKV-immune participants by comparing the neutralizing antibody responses at 1 year post-vaccination for 30 participants with responses at ~8 and ~9 years post-infection for the nine individuals (Figure 4). We found no significant difference in GMT for CHIKV_LR2006_, MAYV_BeAr505411_, and RRV_T-48_ (Figure 4A). For CHIKV_181/25_, the GMT trended ~3.6-fold higher for infection participants compared to vaccinees (* *p* = 0.0361) (Figure 4A). For CHIKV_Brazil-7124_, the GMT trended ~3.1-fold higher for infected participants compared to vaccinees (* *p* = 0.0290) (Figure 4A). For ONNV_UgMP30_, the GMT trended ~9.0-fold higher for infected participants compared to vaccinees (** *p* = 0.0067) (Figure 4A), and we found no significant difference in GMT for CHIKV_LR2006_, ONNV_UgMP30_, MAYV_BeAr505411_, and RRV_T-48_ (Figure 4B). For neutralization against CHIKV_181/25_, the GMT was ~3.0-fold higher for infected participants compared to vaccinees (* *p* = 0.0265) (Figure 4B). The CHIKV_Brazil-7124_ GMT for naturally infected participants was ~3.7-fold higher compared to vaccinees (* *p* = 0.0272) (Figure 4B). Additionally, we examined the Pearson correlation between neutralizing antibody potency and age post-vaccination or post-infection (Supplementary Figure S1). We found no significant correlation between neutralizing antibody titers and age for vaccinees; however, we did detect significant but weak negative correlations between neutralizing antibody titer and age for CHIKV-immune individuals (Supplementary Figure S1). Although neutralization titers were slightly higher in CHIKV-immune individuals for some viruses, we can conclude that IXCHIQ vaccination elicits similar alphavirus neutralizing antibody potency and breadth to CHIKV infection by 1 year post-vaccination.

**Figure 5:**
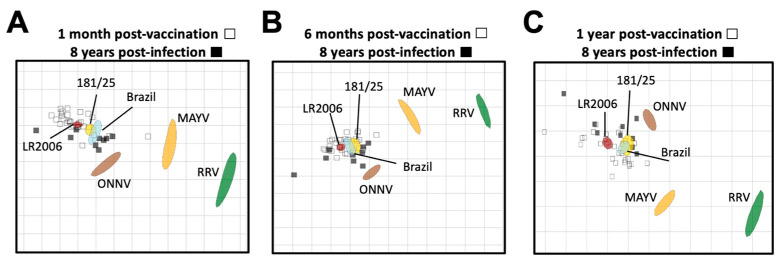
Vaccinee sera cluster antigenically with infection sera. Antigenic cartography was utilized to make an antigenic map to illustrate the relationship between sera and neutralized viruses as well as relative antigen relatedness of the viral antigens compared in this study based on PRNT_50_. The vaccinee sera are represented by unfilled squares and the infection sera at 8 years post-infection are the black filled squares. The viral antigens are plotted as larger blob shapes based on the confidence area of its position. The grids of the antigenic maps are units of antigenic distance and represent a 2-fold change in serum dilution in the neutralization assay. The map is constructed using 50% plaque reduction neutralization titers (PRNT_50_) of each sera sample for each virus which position the sera closest to the viral antigen that was best neutralized, with viruses with lower neutralization potency positioned further away based on calculated pairwise distances between the sera and virus. Antigenic maps are shown comparing sera collected at 8 years post-CHIKV infection to vaccinee serum collected at (**A**) 1 month post-vaccination, (**B**) 6 months post-vaccination, or (**C**) 1 year post-vaccination. The limit of detection for neutralization assays was a PRNT_50_ of 20, and sera falling below the limit of detection are recorded as 10 for calculations of antigenic distances.

We next used neutralization titers from vaccine- and CHIKV infection-immune sera to resolve the antigenic relationships between the alphaviruses using antigenic cartography (Figure 5). Antigenic cartography provides a means to use neutralization titers to graphically visualize antigenic distances between viruses and sera. For this analysis, we utilized the neutralization titers of the vaccinee sera at 1 month, 6 months, and 1 year post-vaccination (Supplemental Table S2), and compared them to the CHIKV natural infection neutralization titers at 8 years post-infection (Supplemental Table S4). In general, neutralization titers placed the vaccine- and CHIKV infection-immune sera in a cluster around the three CHIKV viral strains, comprising a single serogroup with very little difference in antigenic distances between vaccinee and infected samples (Figure 5). Lower neutralization titers against the heterotypic alphaviruses led to more distant placement from all of the sera samples, with ONNV_UgMP30_ being the closest followed by MAYV_BeAr505411_ and then RRV_T-48_, against which all sera had the lowest neutralizing activity (Figure 5). When comparing these antigenic relationships at 1 month post-vaccination and 8 years post-infection, we found that there was about an 8-fold range in antigenic units across the map for all vaccinee and CHIKV infection sera (Figure 5A). When comparing these antigenic relationships at 6 months post-vaccination and 8 years post-infection, the two serum groups clustered even more tightly with about a 6-fold range across the map with the exception of one outlier (Figure 5B). At 1 year post-vaccination, an average of a 6-fold change in antigenic units was also observed with the exception of two outlying sera (Figure 5C). Intriguingly, over time, the viral antigens MAYV and RRV became positioned more closely to the vaccinee sera, suggesting the broadening of cross-neutralizing immunity over time throughout the first year after vaccination (8- to 14-fold change vs. 4- to 12-fold change at 1 year post-vaccination) (Figure 5). Perhaps most importantly, at all timepoint comparisons, we found that the vaccinee sera clustered antigenically with the CHIKV infection-immune sera, suggesting that the IXCHIQ vaccine elicits antibody responses similar to natural CHIKV infection. This conclusion is consistent with our comparative analysis of neutralization titers alone (Figure 4) and supports our conclusion that IXCHIQ vaccination elicits similar cross-neutralizing antibody potency and breadth to CHIKV infection.

## 4. Discussion

Investigators have previously described the alphavirus antibody breadth elicited by CHIKV infection in humans, demonstrating cross-neutralization within the Semliki Forest virus antigenic complex, which is mediated by recognition of similar domains within the envelope proteins [21,22,23,31,32,33,34]. Additionally, the breadth of neutralizing antibodies elicited by other CHIKV vaccines has also been examined in humans [23,35] and mice [36,37,38], but not previously for IXCHIQ (VLA1553, attenuated based on the La Reunion strain of ECSA genotype). Here, we characterized the alphavirus cross-neutralizing antibody responses in humans elicited by the recently approved IXCHIQ vaccine. While Asian genotype CHIKV_181/25_ neutralizing antibody responses elicited by IXCHIQ had been previously reported following NHP studies and Phase 3 trials, this is the first characterization of the human neutralizing antibody response against additional CHIKV strains as well as other related alphaviruses including ONNV_UgMP30_, MAYV_BeAr505411_, and RRV_T-48_ elicited by IXCHIQ.

We found that IXCHIQ vaccination elicits both homotypic and heterotypic neutralizing responses against three strains of CHIKV, ONNV_UgMP30_, MAYV_BeAr505411_, and RRV_T-48_. These responses were durable, with only a modest reduction in neutralization titer between one month and one year post-vaccination. This is important for the populations living in areas with not only CHIKV transmission but also potential for circulation of additional alphaviruses. Given that neutralizing antibodies are a correlate of protection for alphavirus infection in humans [39,40,41], these cross-neutralizing responses have potential to provide cross-protection to populations that are vulnerable to heterotypic alphavirus infection, although increasing antigenic differences between CHIKV and heterotypic alphaviruses may ultimately limit such cross-protective breadth. We found that cross-neutralization decreased in potency with increased phylogenetic distance and Dayhoff distance of amino acid relatedness. Cross-reactive neutralization strongly supports the presence of shared key neutralizing antibody epitopes among the viruses included in our investigation, which we and others have shown to be linked, at least in part, to antibodies targeting the B domain of E2 [21,23,35,42,43,44,45,46]. The small differences in neutralizing activity between the CHIKV strains at all timepoints and a small reduction in PRNT_50_ compared to ONNV_UgMP30_ for the vaccinees and CHIKV-immune participants over all timepoints supports the conclusion that all CHIKV strains cluster as a single serotype [2,3]. Although CHIKV strains cluster antigenically, in some cases, unique amino acid mutations have been demonstrated to contribute to antigenic variation [47], which is why we included a contemporary CHIKV isolate from 2021 in Brazil [24] in this analysis. Ultimately, we found that the mutations in the CHIKV_Brazil-7124_ strain relative to the other CHIKV strains did not greatly impact the neutralization activity of vaccinees or CHIKV-immune individuals against the Brazilian strain. One intriguing result from our study was the modest increases in MAYV_BeAr505411_ and RRV_T-48_ neutralizing activity over time for vaccinees, suggesting that vaccine-elicited cross-reactive immunity broadens over one year post-vaccination; this finding would be consistent with previous work demonstrating that human antibody breadth broadens over time after natural CHIKV infection [21,23]. CHIKV VLP vaccine-elicited (PXVX0317) cross-neutralizing antibodies against ONNV, MAYV, and RRV in humans have been identified by Raju et al., and some of these isolated monoclonal antibodies were shown to partially cross-protect against viral pathogenesis and disease in mice [35]. Our work builds upon these findings of CHIKV vaccine-elicited alphavirus cross-neutralization in humans and reveals the first report of cross-neutralizing antibodies induced by the licensed vaccine IXCHIQ paired with evidence that these antibodies persist at one year post-vaccination and share potency and breadth features consistent with what is seen following natural infection. Additionally, our study directly compares these vaccinee responses to the cross-neutralizing antibodies generated in response to CHIKV infection and shows that IXCHIQ elicits neutralizing antibody populations that are similar in potency and breadth to antibodies elicited by natural CHIKV infection.

While cross-neutralization against other alphaviruses was detected, a limitation of this study is the translational impact that IXCHIQ will have on infection and disease outcomes for these antigenically related alphaviruses. Additionally, future studies are needed to examine the impact of pre-existing alphavirus immunity on the immune response to IXCHIQ when administered to CHIKV pre-immune vaccinees, including the manner in which prior infection can shape the cross-neutralizing antibody breadth. It is possible that prior CHIKV immunity could impact IXCHIQ vaccine efficacy in positive or negative ways. As a live-attenuated virus vaccine, IXCHIQ may be neutralized by pre-exiting CHIKV antibodies in vaccinees with CHIKV infection—a victim of so called “sterilizing immunity”. It is also possible that IXCHIQ vaccination may stimulate the expansion and diversification of latent memory cells from prior infection, giving rise to plasmablasts that may exhibit greater potency and/or breadth than pre-vaccination neutralizing antibodies in vaccinees. These studies have not been carried out to date; rather, Phase III trial participants were screened to select immunologically CHIKV-naïve vaccinees. There is an ongoing trial in adolescent participants in endemic Brazil that will begin to address these questions. Reciprocally, the way that IXCHIQ vaccination can shape the potency and breadth of cross-reactive alphavirus immunity should also be addressed empirically. CHIKV outbreaks have the capacity to be explosive in immunologically naïve populations and then disappear for decades at a time, but it is not known whether CHIKV infection-elicited cross-reactive alphavirus immunity is sufficient to prevent infection with related alphaviruses. While no clinical or epidemiological reports suggest that cross-reactive alphavirus immunity would enhance infection, as has been seen with dengue virus vaccines and earlier RSV vaccine candidates, this question will warrant monitoring as for all new viral vaccines. It is not yet understood how CHIKV seroconversion of endemic populations through immunization could shape the transmission of CHIKV and other alphaviruses. It is also not yet understood how immunization programs will be shaped (universal or targeted) and what the resulting impact on alphavirus transmission will be. To address these open questions, it will be important to characterize the alphavirus cross-neutralizing antibody breadth in individuals with diverse alphavirus exposure histories (vaccinated and unvaccinated) and conduct surveillance for emerging alphaviruses in these regions, especially with related sylvatic transmission cycles. Vaccine rollout in a new population inevitably invites many scientific questions, and it is important that these are urgently addressed to avoid public health risks and ensure that the benefits of vaccination outweigh disease and public health risks. The implementation of the IXCHIQ vaccine and potentially other future approved CHIKV vaccines represents a step forward for the prevention of CHIKV-induced disease burden, impacting millions of people, and has the potential to shape the emergence of additional alphaviruses over the coming years due to cross-reactive immunity.

## Figures and Tables

**Table 1:** Participant demographics for IXCHIQ adult vaccinees and CHIKV-immune individuals.

	IXCHIQ Vaccinees	CHIKV-Immune
Total participants	30	9
Sex		
Female	17 (56.7%)	5 (55.5%)
Male	13 (43.3%)	4 (44.4%)
Country of birth	Not available	Puerto Rico
Country of vaccination or infection	Continental U.S.	Puerto Rico
Age min–max ^1^	19–76	19.5–81.5
Age median ^1^	42.5	38.9
Age standard deviation ^1^	16.12	19.58
Age at vaccination or infection	19–76	12–73
Race		
Hispanic	1 (3.3%)	9 (100%)
Non-Hispanic	29 (96.7%)	0
Not reported	0	0
Unknown	0	0
Ethnicity		
Caucasian/white	25 (83.3%)	2 (22.2%)
Native Hawaiian or Pacific Islander	2 (6.7%)	0
Black or African American	3 (10%)	0
Asian	0	0
Multiracial	0	2 (22.2%)
Other	0	5 (55.5%)
Timepoints for blood draws	Day 1 (baseline), 1 month, 6 months, 1 year post-vaccination	~8 and ~9 years post-infection
Infection symptoms reported		
Asymptomatic		1 (11.1%)
Muscle/joint/bone Pain (severe)		6 (66.6%)
Fever		5 (55.5%)
Headache		4 (44.4%)
Sensitivity to light		5 (55.5%)
Eye pain		3 (33.3%)
Rash		7 (77.7%)
Cough		1 (11.1%)
Runny nose		2 (22.2%)
Malaise		4 (44.4%)
Shortness of breath		1 (11.1%)
Loss of appetite		4 (44.4%)
Diarrhea		1 (11.1%)

^1^ Age in this context refers to participant age at first blood draw when the study was initiated.

**Table 2:** Summary of alphavirus neutralizing antibody responses in all participants.

PRNT_50_	IXCHIQ Vaccinees			CHIKV-Immune (Puerto Rican Cohort)	
CHIKV_LR2006_	1 Month	6 Months	1 Year	~8 Years	~9 Years Post-Infection
Min–Max	34,333–109,913	1363–38,895	728–177,237	4091–64,794	4297–50,825
Geometric mean	16,022	5197	5147	17,317	12,530
Mean	23,181	7140	12,983	25,901	16,863
Standard deviation	24,954	7379	32,100	21,826	15,639
Participants that seroconverted (PRNT_50_ ≥ 20)	30 (100%)	30 (100%)	30 (100%)	9 (100%)	9 (100%)
CHIKV_181/25_					
Min–Max	1619–91,693	1078–13,632	859–15,395	3578–52,100	2766–30,003
Geometric mean	10,879	3714	3440	12,522	10,146
Mean	22,489	4681	4370	17,850	13,126
Standard deviation	28,393	3492	3131	16,069	9664
Participants that seroconverted (PRNT_50_ ≥ 20)	30 (100%)	30 (100%)	30 (100%)	9 (100%)	9 (100%)
CHIKV_Brazil-7124_					
Min–Max	1619–34,109	774–18,838	1124–19,412	3552–35,772	4504–47,026
Geometric mean	6491	3881	3792	11,639	14,138
Mean	8305	5110	5085	15,039	19,465
Standard deviation	6728	4188	4266	11,202	15,978
Participants that seroconverted (PRNT_50_ ≥ 20)	30 (100%)	30 (100%)	30 (100%)	9 (100%)	9 (100%)
ONNV_UgMP30_					
Min–Max	259–8395	299–5800	240–4026	3771–30,296	3505–75,454
Geometric mean	1676	1102	1156	10,370	10,360
Mean	2122	1361	1435	13,206	19,300
Standard deviation	1648	1067	946	9751	24,097
Participants that seroconverted (PRNT_50_ ≥ 20)	30 (100%)	30 (100%)	30 (100%)	9 (100%)	9 (100%)
MAYV_BeAr505411_					
Min–Max	35.5–6259	128–2110	145–5863	194–14,652	470–40,903
Geometric mean	374	417	652	1462	2471
Mean	954	524	967	2860	6797
Standard deviation	1737	410	1158	4491	12,926
Participants that seroconverted (PRNT_50_ ≥ 20)	30 (100%)	30 (100%)	30 (100%)	9 (100%)	9 (100%)
RRV_T-48_					
Min–Max	10–12,271	10–571	10–232.5	25.4–675	29.9–651
Geometric mean	31.7	32.5	39.25	135	112
Mean	444	54.7	56.6	206	166
Standard deviation	2234	101	51.9	198	190
Participants that seroconverted (PRNT_50_ ≥ 20)	20 (66.6%)	25 (83.3%)	25 (83.3%)	9 (100%)	9 (100%)

Note: undetectable PRNT_50_ is recorded as 10.

## Data Availability

The original contributions presented in the study are included in the article/Supplementary Materials; further inquiries can be directed to the corresponding author.

## Supplementary Materials

The following supporting information can be downloaded at https://www.mdpi.com/article/10.3390/vaccines12080893/s1: Figure S1: Correlating age and antibody titer after infection or vaccination; Table S1: Viral stock sequencing analysis summary for the alphaviruses used in this study; Table S2: Compiled raw neutralization titers for vaccinee participants; Table S3: Amino acid sequences (E1/6K/E2/E3) used for phylogenetic and Dayhoff distance analyses to compare the genetic relatedness of the alphaviruses under investigation in this study; Table S4: Compiled raw neutralization titers for CHIKV infection participants.
