# Supplementary material for: The Approved Live-Attenuated Chikungunya Virus Vaccine (IXCHIQ®) Elicits Cross-Neutralizing Antibody Breadth Extending to Multiple Arthritogenic Alphaviruses Similar to the Antibody Breadth Following Natural Infection
Source: Vaccines (Basel). 2024 Aug 7;12(8):893. doi: 10.3390/vaccines12080893 (PMC11359099; doi:10.3390/vaccines12080893)
Supplement: Supplementary file 1 [file vaccines-12-00893-s001.zip › vaccines-3117746-supplementary.pdf]

vaccines

MDPI

The approved live-attenuated chikungunya virus vaccine (IXCHIQ®) elicits cross-neutralizing antibody breadth extending to multiple arthritogenic alphaviruses similar to the antibody breadth following natural infection

Supplemental Figure S1 and Tables S1–S4

7.5.24

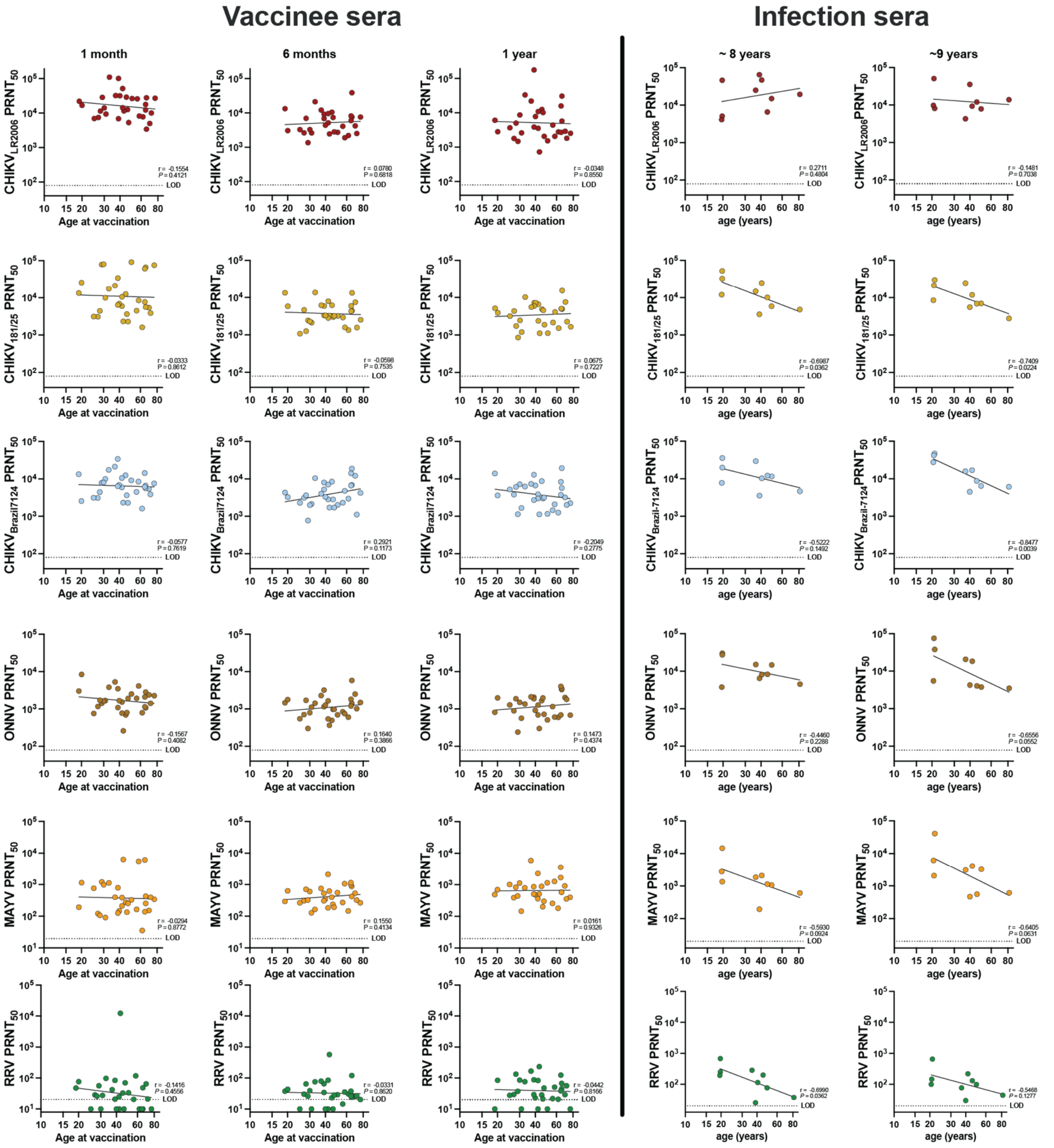

Supplemental Figure S1. Correlating age and antibody titer after vaccination or infection. Virus-specific 50% plaque reduction neutralization titer (PRNT$_{50}$) and age at vaccination or age at blood draw after infection are plotted. Age and PRNT$_{50}$ are log-transformed and analyzed by Pearson correlation. Pearson correlation coefficients (r) and corresponding *P* values are shown with a simple linear regression fit to the log-transformed data. Variable slope, non-linear regressions in Prism software were used to calculate

$PRNT_{50}$. The limit of detection for neutralization assays was 20 and sera falling below the limit of detection are assigned a value of 10.

Supplemental Table S1. Viral stock sequencing analysis summary for the alphaviruses used in this study.

Virus	Position	Nucleotide Change	A.A. Change	Variant Frequency	Region	# of Virus Reads / Total Reads	Virus Reference Sequence
**CHIKV Brazil 7124**	688	G -> K	Arg -> Met	55.80%	NSP 1	1,354,589 of 4,624,952	CHIKV TO-UFT-7124 Accession: ON586955.1
	691	A -> G	No Change	35.60%	NSP 1		
	1361	G -> A	No Change	54.40%	NSP 1		
	9083	A -> C	No Change	56.30%	E2B domain		
**CHIKV 181/25**	3706	G -> A	Arg -> His	98.70%	NSP 1	344,119 of 5,133,734	CHIKV 181/25 Accession: MW473668.1
	8345	A -> G	No Change	39.10%	NSP 2		
**CHIKV LR2006 OPY1**	561	A -> G	Gln -> Arg	98.40%	NSP 1	2,800,692 of 3,517,670	CHIKV LR2006 OPY1 Accession: KY575571.1
	1025	G -> A	Val -> Met	95.50%	NSP 1		
	4140	A -> G	Asp -> Gly	93.10%	NSP 3		
**MAYV BeAr505411**	1362	G -> C	Lys -> Asn	85.9	NSP 1-3	2,007,089 of 2,857,853	MAYV BeAr505411 Accession: KP842818.1
	3980	C -> A	Pro ->Gln	99.8	NSP 1-3		
	9501-9503	Deletion of CAG	Gln	100	E2		
**ONNV UGMP30**	173	G -> A	Val -> Ile	25.00%	NSP 1	38,028 of 2,635,751	ONNV UgMP30 Accession: M20303.1
	957	G -> A	Ser -> Asn	100.00%	NSP 1		
	1008	G -> A	Gly -> Asp	100.00%	NSP 1		
**RRV T-48**	314	T -> A	Cys -> Ser	99.80%	NSP 1	748,882 of 4,585,162	RRV T48 Accession: GQ433359.1
	413	T -> G	Ser -> Ala	99.50%	NSP 1		
	1138	T -> C	No Change	99.80%	NSP 1		
	1466	A -> C	Ile -> Leu	99.70%	NSP 1		
	5085	G -> T	Ser -> Ile	97.90%	NSP 3		
	5263	T -> C	No Change	99.40%	NSP 3		
	7736	Y -> C	No Change or Ser -> Phe	97.10%	Capsid		
	8585	A -> T	No Change	25.40%	E2		
	8623	Y -> T	No Change	97.70%	E2		
	8766	R -> G	Gln -> Arg or No Change	98.80%	E2		

Supplemental Table S2. Compiled raw neutralization titers for vaccinee participants.

	CHIKV$_{LR2006}$				CHIKV$_{181/25}$			
**Participant**	**Day 1**	**Day 29**	**Day 180**	**Day 365**	**Day 1**	**Day 29**	**Day 180**	**Day 365**
1	10	101155	9166	177237	10	34109	3316	7191
2	10	29029	10538	12578	10	12484	6201	4867
3	10	9402	2611	2611	10	9960	2123	1194
4	10	12627	7373	10248	10	3651	3001	4542
5	10	11561	2450	728.9	27.02	2328	3266	1113
6	10	14237	5155	10876	10	5967	2827	2367
7	10	25999	4086	4388	10	4485	2928	2148
8	10	27816	38895	30689	10	66596	13244	15395
9	10	109913	21270	33314	22.66	17445	13642	10241
10	10	51572	9610	3540	10	10538	7917	6585
11	10	22011	13297	6094	10	13351	13392	5170
12	10	31911	4393	7825	10	6935	3312	7229
13	10	16756	3053	2828	10	25713	5846	3908
14	10	5001	4205	2910	10	5386	7500	7590
15	10	7467	2629	1794	10	3092	4635	4363
16	10	3433	7574	5736	10	5599	4431	4915
17	10	6863	6846	3857	30.67	6392	5913	5901
18	10	28583	1363	1480	10	78606	2432	859.2
19	10	27071	7609	2543	10	75997	2524	1654
20	10	7801	2209	2785	10	1619	1342	1523
21	10	8220	1894	2073	10	8467	1587	4096
22	10	32292	12139	21936	33.95	21182	6191	5544
23	10	12327	6022	2706	10	61193	5936	5005
24	10	17793	8194	15923	10	7696	4297	5069
25	10	26534	2739	1533	10	91693	3216	1118
26	10	14219	3274	8661	22.23	80449	2288	2454
27	10	11465	6993	5019	10	4427	1258	1739
28	10	6967	3285	3673	10	3139	1078	3176
29	10	5339	2804	2076	10	2298	3220	1870
30	10	10053	2532	1816	10	3879	1571	2269

	CHIKV$_{Brazil-7124}$				ONNV$_{UgMP30}$			
**Participant**	**Day 1**	**Day 29**	**Day 180**	**Day 365**	**Day 1**	**Day 29**	**Day 180**	**Day 365**
1	10	34109	8015	12962	10	3477	1149	2070
2	10	12484	8448	8771	10	690.4	495.2	1671
3	10	9960	2135	1670	22.45	1677	1118	564
4	10	3651	2830	3002	10	796.7	973.9	1978
5	10	2328	1714	1124	10	259.3	365.3	300.6
6	10	5967	2845	3874	10	769.9	538.3	690.6
7	10	4485	2314	1451	10	1928	838.9	1155
8	10	6659	18838	19412	25.84	2828	5800	3307
9	10	17445	10480	6273	29.3	3814	1422	1357

10	10	10538	7041	3029	30.03	1533	1645	1266
11	10	13351	4325	14015	27.82	3023	1464	811.6
12	10	6935	3306	6711	35.44	1673	1271	915.1
13	10	2571	3261	3616	44.96	8395	1784	1982
14	10	5386	12094	3203	10	2383	2492	2033
15	10	3092	3122	3831	10	1165	706.7	889.9
16	10	5599	6913	5740	10	1073	1404	3514
17	10	6392	5227	2714	41.22	1089	3206	1873
18	10	7860	774.1	1140	10	1418	299.1	240.4
19	10	7599	4246	2245	10	2260	1492	684.4
20	10	1619	2959	1260	10	792.9	602.9	594.1
21	10	8467	4808	2092	10	4142	746.5	606.5
22	10	21182	4018	7400	10	5291	2276	2114
23	10	6119	14147	3750	25.71	1140	1819	691.2
24	10	14387	6644	8373	10	2091	1193	4026
25	10	9169	2018	1168	44.45	2519	695.3	510.3
26	10	8044	2001	7193	10	1624	791.8	1505
27	10	4427	3658	6111	10	1716	1705	1874
28	10	3139	2302	5240	10	757.6	544.8	1288
29	10	2298	1711	3171	23.5	1910	978.2	720.5
30	10	3879	1112	2013	10	1413	1016	1807

	$MAYV_{BeAr505411}$				RRV_{T48}			
**Participant**	**Day 1**	**Day 29**	**Day 180**	**Day 365**	**Day 1**	**Day 29**	**Day 180**	**Day 365**
1	10	791.4	528.4	862.3	10	83.56	65.03	87.96
2	10	276.1	401.1	880	10	32.81	23.02	28.57
3	10	90.82	128.1	144.6	10	27.37	10	10
4	10	134.5	237	200.4	10	10	10	21.18
5	10	6295	625.2	507.5	10	12271	571.1	232.5
6	10	210.8	184.7	357.6	10	10	10	10
7	10	164.2	271.7	256	10	20.52	14.48	21.66
8	10	427.1	871.2	575.9	10	48.16	120.6	45.11
9	10	1128	163.6	792.3	10	97.43	75.81	76.37
10	10	387.9	2110	2269	10	24.93	85.05	83.43
11	10	193	294.6	485	31.81	47.7	37.62	10
12	10	147.4	346.6	481.3	10	10	10	22.85
13	10	1147	633.9	692.6	10	76.45	43.21	84.71
14	10	152.4	533.6	910.8	10	64.73	25.35	28.92
15	10	121.7	308.4	388.9	10	28.52	30.21	29.34
16	10	254.7	423.2	572.6	10	10	23.75	67.28
17	10	126.4	308.2	504.8	10	20.88	34.28	29.16
18	10	1205	324.9	438.1	10	56.1	25.91	37.33
19	10	343.4	266.3	398	10	10	20.18	10
20	10	35.56	147.6	180.4	10	10	30.67	20.65

21	10	5521	1070	1260	10	117.4	35.34	50.73
22	10	395.5	754	5863	10	42.01	79.29	167.9
23	10	138.1	985.4	1679	10	10	26.01	38.34
24	22.26	6115	1149	3553	10	10	37.42	124.6
25	10	326.3	555.9	1148	10	33.32	29.03	10
26	10	978.5	604.4	1115	10	10	38.98	85.57
27	10	106.7	710.1	821	10	25.98	64.86	133.1
28	10	761.8	266.1	1012	10	10	10	27.69
29	10	254.9	193.3	298	10	69.74	28.08	47.4
30	10	398	324.1	353.3	10	20.97	26.86	56.17

Supplemental Table S3. Amino acid sequences (E1/6K/E2/E3) used for phylogenetic and Dayhoff distance analyses to compare the genetic relatedness of the alphaviruses under investigation in this study.

**CHIKV LR2006-OPY1**<br>SLAIPVMCLLANTTFPCSQPPCTPCCYEKEPEETLRM-<br>LEDNVMRPGYYQLLQASLTCSPHRQRRSTKDNFNVYKATRPYLAHCPDCGEGHSCHSPVALERIRN<br>EATDGTLKIQVSLQIGIKTDDSHDWTKLRYMDNHMPADAERAGLFVRTSAPCTITGTMGH-<br>FILARCPKGETLTVGFTDSRKISHSCTHPFHHDPPVIGREKFHSRPQHGKELPCSTYVQSTAATTEEIEV<br>HMPPDTPDRTLMSQQSGNVKITVNGQTVRYKCNCGGSNEGLTTTDKVINNCKVDQCHAA-<br>VTNHKKWQYNSPLVPRNAELGDRKGKIHIPFPLANVTCRVPKARNPTVTYGKNQVIMLLYPDHPTL<br>LSYRNMGEEPNYQEEWVMHKKEVVLTVPTEGLEVTWGNNEPYKYWPQLSTNG-<br>TAHGHPHEIILYYYELYPTMTVVVVSVATFILLSMVGMAAGMCMCARRRCITPYELTPGATVPFLLSL<br>ICCIRTAKAATYQEAAIYLWNEQQPLFWLQALIPLAALIVLCNCLRLL-<br>PCCCKTLAFLAVMSVGAHTVSAYEHVTVIPNTVGVPYKTLVNRPGYSPMVLEMELLSVTLEPTLSLD<br>YITCEYKTVIPSPYVKCCGTAECKDKNLPDYSCKVFTGVYPFMWGGAYCFCDAENTQLSEAH-<br>VEKSESCKTEFASAYRAHTASASAKLRVLYQGNNITVTAYANGDHAVTVKDAKFIVGPMSSAWTPF<br>DNKIVVYKGDVYNMDYPPFGAGRPGQFGDIQSRTPESKDVYANTQLVLQRPAVGTVHVPYS-<br>QAPSGFKYWLKERGASLQHTAPFGCQIATNPVRAVNCAVGNMPISIDIPEAAFTRVVDAPSLTDMSC<br>EVPACTHSSDFGGVAIIKYAASKKGKCAVHSMTNAVTIREAEIEVEGNSQLQISFSTA-<br>LASAEFRVQVCSTQVHCAAECHPPKDHIVNYPASHTTLGVQDISATAMSWVQKITGGVGLVVAVA<br>ALILIVVLCVSFSRH
**CHIKV 181/25**<br>SLAIPVMCLLANTTFPCSQPPCTPCCYEKEPEKTLRM-<br>LEDNVMSPGYYQLLQASLTCSPRRQRRSIKDNFNVYKAIRPYLAHCPDCGEGHSCHSPVALERIRNE<br>ATDGTLKIQVSLQIGIKTDDSHDWTKLRYMDNHMPADAERARLFVRTSAPCTITGTMGH-<br>FILARCPKGETLTVGFTDGRKISHSCTHPFHHDPPVIGREKFHSRPQHGRELPCSTYAQSTAATAEEIE<br>VHMPPDTPDRTLMSQQSGNVKITVNSQTVRYKCNCGDSNEGLTTTDKVINNCKVDQCHAA-<br>VTNHKKWQYNSPLVPRNAELGDRKGKVHIPFPLANVTCRVPKARNPTVTYGKNQVIMLLYPDHPT<br>LLSYRNMGEEPNYQEEWVTHKKEIRLTVPTEGLEVTWGNNEPYKYWPQLSTNG-<br>TAHGHPHEIILYYYELYPTMTVVVVSVASFVLLSMVGVAVGMCMCARRRCITPYELTPGATVPFLLSL<br>ICCIRTAKAATYQEAAVYLWNEQQPLFWLQALIPLAALIVLCNCLRLLPCFCKTLT-<br>FLAVMSVGAHTVSAYEHVTVIPNTVGVPYKTLVNRPGYSPMVLEMELLSVTLEPTLSLDYITCEYKTV<br>IPSPYVKCCGTAECKDKSLPDYSCKVFTGVYPFMWGGAYCFCDTENTQLSEAH-<br>VEKSESCKTEFASAYRAHTASASAKLRVLYQGNNVTVSAYANGDHAVTVKDAKFIVGPMSSAWTPF<br>DNKIVVYKGDVYNMDYPPFGAGRPGQFGDIQSRTPESEDVYANTQLVLQRPSAGTVHVPYS-<br>QAPSGFKYWLKERGASLQHTAPFGCQIATNPVRAMNCAVGNMPISIDIPDAAFTRVVDAPSLTDMS

CEVPACTHSSDFGGVAIIKYAASKKGKCAVHSMTNAVTIREAEIEVEGNSQLQISFSTA-<br>LASAEFRVQVCSTQVHCAAECHPPKDHIVNYPASHTTLGVQDISVTAMSWVQKITGGVGLVVAVA<br>ALILIVVLCVSFSRH
**CHIKV Brazil-7124**<br>SLAIPVMCLLANTTFPCSQPPCTPCCYEREPEETLRM-<br>LEDNVMRPGYYQLLQASLTCSPHRQRRSTKDNFNVYKATRPYLAHCPDCGEGHSCHSPVALERIRN<br>EATDGTLKIQVSLQIGIKTDDSHDWTKLRYMDNHTPADAERAGLFVRTSAPCTITGTMGH-<br>FILTRCPKGETLTVGFTDSRKISHSCTHPFHHDPPVIGREKFHSRPQHGKELPCSTYVQSTAATTEEIEV<br>HMPPDTPDRTLMSQQSGNVKITVNGQTVRYKCNCGGSNEGLITTDKVINNCKVDQCHAA-<br>VTNHKKWQYNSPLVPRNAELGDRKGKIHIPFPLANVTCRVPKARNPTVTYGKNQVIMLLYPDHPTL<br>LSYRNMGEEPNYQEEWVTHKKEVVLTVPTEGLEVTWGNNEPYKYWPQLSTNG-<br>TAHGHPHEIILYYYELYPTMTVVVVSVASFVLLSMVGVAVGMCMCARRRCITPYELTPGATVPFLLSL<br>ICCIRTAKAATYQEAAVYLWNEQQPLFWLQALIPLAALIVLCNCLRLL-<br>PCCCKTLAFLAVMSVGAHTVSAYEHVTVIPNTVGVPYKTLVNRPGYSPMVLEMELLSVTLEPTLSLD<br>YITCEYKTVIPSPYVKCCGTAECKDKNLPDYSCKVFTGVYPFMWGGAYCFCDAENTQLSEAH-<br>VEKSESCKTEFASAYRAHTASASAKLRVLYQGNNITVTAYANGDHAVTVKDAKFIVGPMSSAWTPF<br>DNKIVVYKGDVYNMDYPPFGAGRPGQFGDIQSRTPESTDVYANTQLVLQRPAAGTVHVPYS-<br>QAPSGFKYWLKERGASLQHTAPFGCQIATNPVRAMNCAVGNMPISIDIPDAAFIRVVDAPSLTDMSC<br>EVPTCTHSSDFGGVAIIKYAASKKGKCAVHSMTNAVTIREAEIEVEGNSQLQISFSTA-<br>LASAEFRVQVCSTQVHCVAECHPPKDHIVNYPASHTTLGVQDISATALSWVQKITGGVGLVVAVAA<br>LILIVVLCVSFSRR
**ONNV-UgMP30**<br>SLALPVMCLLANTTFPCSQPPCAPCCYEKKPEETLRM-<br>LEDNVMQPGYYQLLDSALACSQRRQKRNARENFNVYKVTRPYLAHCPDCGEGHSCHSPIALERIRS<br>EATDGTLKIQVSLQIGIKTDDSHDWTKLRYMDSHTPVDADRSGLFVRTSAPCTITGTMGH-<br>FILARCPKGETLTVGFVDSRRISHTCMHPFRHEPPLIGREKFHSRPQHGKELPCSTYVHTTAATAEEIE<br>VHMPPDTPDYTLMTQQAGNVKITVDGQTVRYKCKCDGSNEGLITADKVINNCK-<br>VDQCHTAVTNHKKWQYNSPLTPRNSEQGDRKGKIHIPFPLVNTTCRVPKARNPTVTYGKNRVTLLL<br>HPDHPTLLSYRAMGRIPDYHEEWITNKKEISITVPAEGLEVTWGNNDPYKYWPQLSTNG-<br>TAHGHPHEIILYYYELYPTTTIAVLAAASIVITSLVGLSLGMCICARRRCITPYELTPGATIPFLLGVLCC<br>ARTAKAASYYEAATYLWNEQQPLFWLQLLIPLSAAIVVCNCLKLLPCCCKTLT-<br>FLAVMSIGARTVTAYEHATVIPNTVGVPCKTLVSRPGYSPMVLEMELQSVTLEPALSLDYITCEYKTIT<br>PSPYVKCCGTAECKAKNLPDYNCKVFTGVYPFMWGGAYCFCDAENTQLSEAH-<br>VEKSESCKTEFASAYRAHTASVSAKLRVFYQGNNITVSAYANGDHAVTVEDAKFVIGPLSSAWSPFD<br>NKIVVYKGEVYNMDYPPFGAGRPGQFGDIQSRTPDSKDVYANTQLILQRPAAGAIHVPYS-<br>QAPSGFKYWLKEKGASLQHTAPFGCQIATNPVRAVNCAVGNIPVSIDIPDAAFTRVTDAPSITDMSC<br>EVASCTHSSDFGGAAVIKYTASKKGKCAVHSVTNAVTIREPNVDVKGTAQLQIAFSTA-<br>LASAEFKVQICSTLVHCSATCHPPKDHIVNYPSPHTTLGVQDISTTAMSWVQKITGGVGLVVAIAALI<br>LIIVLCVSFSRH
**MAYV-BeAr505411**<br>ASTVTAMCLLTNISFPCFQPSCAPCCYEKGPEPTLRMLEENVNSEGYYDLLHAA-<br>VYCRNSSRSKRSTANHFNAYKLTRPYVAYCADCGMGHSCHSPAMIENIQADATDGTLKIQFASQIGL<br>TKTDTHDHTKIRYAEGHDIAEAARSTLKVHSSSECTVTGTMGHFILAKCPPGEAISVSFVDS-<br>KNEHRTCRIAYHHEQRLIGRERFTVRPHHGIELPCTTYQLTTAETSEEIDMHMPPDIPDRTILSQQSGN<br>VKITVNGRTVRYSCSCGSKPSGTTTTDKTINSCTVDKCQAYVTSHTKWQFNSPFVPRAE-<br>QAERKGKVHIPFPLINTTCRVPLAPEALVRSGKREATLSLHPIHPTLLSYRTLGAEPVFDEQWITAQTE<br>VTIPVPVEGVEYQWGNHKPQRLWSQLTTEGKAHGWPHEIIEYYY-<br>GLHPTITIVVVIAVSVVVLLSLAASVYMCVVARNKCLTPYALTPGAVVPVTIGVLCCAPKAHAASFAE<br>GMAYLWDNNQSMFWMELTGPLALLILTTCCARSLLSCCKGSFLVAMSIGSAVASAYEH-<br>TAIIPNQVGFPYKAHVAREGYSPLTLQMQVVETSLEPTLNLEYITCDYKTKVPSPYVKCCGTAECRTQ

DKPEYKCAVFTGVYPFMWGGAYCFCDSENTQMSEAYVERADVCKHDHAAAYRAHTASLRA-<br>QIKVTYGTVNQTVEAYVNGDHAVTIAGTKFIFGPVSTAWTPFDTKIVVYKGEVYNQDFPPYGAGQP<br>GRFGDIQSRTLDSRDLYANTGLKLARPAAGNIHVPYTQTPSGFKTWQKDRDSPLNAKAP-<br>FGCVIQTNPVRAMNCAVGNIPVSMDIADSAFTRLTDAPVISELTCTVSTCTHSSDFGGIAVLSYKVEKP<br>GRCDIHSHSNVAVLQEVSIETEGRSVIHF-<br>STASAAPSFVVSVCSSRATCTAKCEPPKDHVVTYPANHNGVTLPDLSSTAMTWAQHLAGGVGLLIV<br>LAVLILVIVTCVTLRR
**RRV-T-48**<br>WSAALMMCILANTSFPCSSPPCYPCCYEKQPEQTLRMLEDNVNRPGYYELLEASMTCRNRS-<br>RHRRSVTEHFNVYKATRPYLAYCADCGDGYFCYSPVAIEKIRDEASDGMLKIQVSAQIGLDKAGTHA<br>HTKIRYMAGHDVQESKRDSLRVYTSAACSIHGTMGHFIVAHCPPGDYLKVSFEDAD-<br>SHVKACKVQYKHDPLPVGREKFVVRPHFGVELPCTSYQLTTAPTDEEIDMHTPPDIPDRTLLSQTAG<br>NVKITAGGRTIRYNCTCGRDNVGTTSTDKTINTCKIDQCHAA-<br>VTSHDKWQFTSPFVPRADQTARRGKVHVPFPLTNVTCRVPLARAPDVTYGKKEVTLRLHPDHPTLF<br>SYRSLGAEPHPYEEWVDKFSERIIPVTEEGIEYQWGNNPPVRLWAQLTTE-<br>GKPHGWPHEIIQYYYGLYPAATIAAVSGASLMALLTLAATCCMLATARRKCLTPYALTPGAVVPLTL<br>GLLCCAPRANAASFAETMAYLWDENKTLFWMEFAAPAAALALLACCIKSLICCCK-<br>PFSFLVLLSLGASAKAYEHTATIPNVVGFPYKAHIERNGFSPMTLQLEVVETSLEPTLNLEYITCEYKTV<br>VPSPFIKCCGTSECSSKEQPDYQCKVYTGVYPFMWGGAYCFCDSENTQLSEAYVDRSDVCK-<br>HDHASAYKAHTASLKATIRISYGTINQTTEAFVNGEHAVNVGGSKFIFGPISTAWSPFDNKIVVYKDD<br>VYNQDFPPYGSGQPGRFGDIQSRTVESKDLYANTALKLSRP-<br>SPGVVHVPYTQTPSGFKYWLKEKGSSLNTKAPFGCKIKTNPVRAMDCAVGSIPVSMDIPDSAFTRVV<br>DAPAVTDLSCQVVVCTHSSDFGGVATLSYKTDKPGKCAVHSHSNVATLQEAT-<br>VDVKEDGKVTVHFSTASASPAFKVSVCDAKTTCTAACEPPKDHIVPYGASHNNQVFPDMSGTAMT<br>WVQRLASGLGGLALIAVVVLVLVTCITMRR

Supplemental Table S4. Compiled raw neutralization titers for CHIKV infection participants.

Years post-infection	Partici-pant,visit	CHIKV LR2006-OPY1	CHIKV 181/25	CHIKV Brazil-7124	ONNV UgMP30	MAYV BeAr505411	RRV T-48
8	70001 v1	46285	52100	35772	30296	14652	674.6
9.3	70001 v2	8114	30003	47026	37990	40903	651.4
8.15	70003 v1	64794	3578	3552	6496	193.6	25.4
9.2	70003 v2	35561	5546	4504	4266	469	29.92
8.15	70005 v1	5093	32735	19960	27464	1355	250.1
9.19	70005 v2	50825	21174	42058	75454	2061	146.4
8.19	70008 v1	4091	11932	7765	3771	2787	194.0
9.19	70008 v2	9785	8458	27542	5498	6095	99.95
7.81	70011 v1	46829	24822	10302	8132	2092	113.9
8.96	70011 v2	9322	11792	16920	18421	4114	218.0
8.2	70012 v1	25074	14730	29663	15089	1860	283.6
8.77	70012 v2	4297	24603	15878	20747	3101	76.86
8.12	70013 v1	14929	5881	11602	14772	1059	76.86
8.74	70013 v2	7897	6958	6418	3769	3261	98.63
8.28	70015 v1	19441	4800	4642	4516	609.5	37.34
8.75	70015 v2	13890	2766	6118	3505	615.6	44.51
8.28	70016 v1	6576	10075	12091	8315	1131	198.4
8.75	70016 v2	12072	6848	8718	4050	556.3	130.5
